# Inter-Cohort Cannibalism of Early Benthic Phase Blue King Crabs (*Paralithodes platypus*): Alternate Foraging Strategies in Different Habitats Lead to Different Functional Responses

**DOI:** 10.1371/journal.pone.0088694

**Published:** 2014-02-18

**Authors:** Benjamin Daly, W. Christopher Long

**Affiliations:** Kodiak Laboratory, Resource Assessment and Conservation Engineering Division, Alaska Fisheries Science Center, National Marine Fisheries Service, National Oceanic and Atmospheric Administration, Kodiak, Alaska, United States of America; North Carolina State University, United States of America

## Abstract

Blue king crabs (*Paralithodes platypus*) are commercially and ecologically important in Alaska, USA, but population abundances have fluctuated over the past several decades likely resulting from a combination of environmental and biological factors, including recruitment variability. Cannibalism between cohorts may be a source of mortality limiting recruitment success in the wild, but the degree of inter-cohort cannibalism is unknown for early benthic phase blue king crabs. In laboratory experiments, we evaluated the effects of habitat type (sand and shell) on the predator functional response and foraging behavior of year-1 blue king crabs as predators of year-0 conspecifics and examined the effects of predator presence on crypsis of prey crabs. In sand, consumption rates increased with predator size and prey density until satiation, while predation rates in shell were low regardless of predator size or prey density. These differential predation rates yielded a type III functional response in sand but a type I functional response in shell habitat. Crypsis of prey crabs was generally high and did not change in the presence of predators. Predator foraging activity was reduced in shell and may be an adaptive behavior to balance foraging efficiency and susceptibility to larger predators. Our results demonstrate that early benthic phase blue king crabs are cannibalistic between cohorts in the laboratory and that shell material is extremely effective for reducing encounter rates with conspecific predators. The distribution and abundance of such habitat may be important for recruitment success in some populations. Future studies should compare benthic habitat and species assemblages in areas with variable abundances, such as the Pribilof Islands and Saint Matthew Island in the eastern Bering Sea, to better understand possible mechanisms for recruitment variability.

## Introduction

Cannibalism is widespread in the animal kingdom and occurs among both vertebrates and invertebrates [1], [2]. Stomach content analysis and field surveys have documented cannibalism in various decapod crustacean species in the wild [3]–[9] showing that it is a natural behavior. Cannibalism may be intense during periods of strong recruitment and may influence population dynamics of some crustacean species [6], [7], [10]–[12]. For example, older juveniles consume settling post-larvae and recently-settled juveniles, reducing year-class strength of grapsid crab (*Hemigrapsus penicillatus*) [4], Dungeness crab (*Cancer magister*) [6], snow crab (*Chionoecetes opilio*) [7], common Chilean predatory crab (*Acanthocyclus gayi*) [12], and blue crab (*Callinectes sapidus*) [13].

An important aspect of predator-prey relationships is the predator functional response which describes how predation varies with prey density [14], [15]. Three common types of functional responses include type I or density-independent, type II or inversely density-dependent, and type III or density-dependent. The functional response can determine the stability of predator-prey relationships and whether prey persistence is possible; type II functional responses can be destabilizing to predator-prey relationships because proportional predation rates are highest at low prey densities, whereas type III functional responses are stabilizing as predation rates are lowest at low prey densities[16]. The functional response can be changed by a number of factors including habitat (e.g., [17]–[19]), the presence of alternative prey [20], [21], predator size [22], prey size [23], and the spatial arrangement of prey patches [24], [25].

The commercially and ecologically important blue king crab (*Paralithodes platypus*) occurs in isolated populations throughout the North Pacific including waters off Alaska, USA, Japan, and Russia. Commercial fisheries were developed in Alaskan waters around the Pribilof Islands and Saint Matthew Island in the 1970s and peaked in the early 1980s, but population declines caused fishery closures in both areas in the late 1990s [26]. The Saint Matthew stock was declared rebuilt in 2009 and briefly reopened to commercial fishing until low abundance estimates caused a fishery closure in 2013. The Pribilof stock remains closed to commercial fishing today because of extremely low population abundance estimates [26], [27]. Reasons for the population fluctuations are unclear, but large-scale processes such as recruitment variability are likely at play.

Blue king crabs have a complex life cycle, including four pelagic larval stages, a semi-benthic post-larval stage, and benthic juvenile and adult stages. Although we have a basic understanding of the blue king crab life cycle, we know little about its ecology, particularly during the early benthic phase (approximately age 0–2 years). Like the related red king crab (*P. camtschaticus*), early benthic phase blue king crabs are solitary and cryptic with a strong affinity for habitats with complex physical structures [28]–[31], which mediates vulnerability to some predators [32], [33]. Field surveys indicate early benthic phase blue king crabs prefer shell hash [28]–[31], and the relatively smooth carapace suggests a reliance on spatial avoidance as an anti-predator mechanism [33], rather than spination as a predator defense such as with red king crab.

Relatively little is known about the degree of cannibalism in juvenile blue king crabs. Cannibalism occurs in the laboratory for juvenile red king crabs within and between cohorts [34]–[37], although recently-settled, year-0 blue king crabs display low incidence of cannibalism compared to year-0 red king crabs reared under identical conditions [38], [39]. Broader size differences associated with different juvenile cohorts would likely exacerbate cannibalism, yet the degree of cannibalism between year classes remains untested. Field studies suggest blue king crab cannibalism occurs in the wild [40] and the spatial overlap of year-1 and year-0 individuals around the Pribilof Islands [29] implies that cannibalism between cohorts may be a source of mortality.

Stock enhancement through the release of cultured juveniles has been proposed as a possible recovery tool for the depressed Pribilof blue king crab population. Hatchery rearing techniques have been established for larval and juvenile red king crabs [34], [41], [42] but are less developed for blue king crabs (but see [39], [43]). Understanding cannibalistic behavior of early benthic phase blue king crabs will help refine hatchery rearing techniques and develop optimal release strategies. For example, high levels of inter- and intra-cohort cannibalism among early benthic phase red king crabs indicate out-stocking efforts should target complex habitats and release crabs at low densities in a given area once every two years to reduce predation by larger conspecifics [35] as juveniles begin to display social aggregative behavior (podding) and move out of most complex habitats during the second year [44].

Our study was designed to evaluate inter-cohort cannibalism of early benthic phase blue king crabs. To meet this objective, we examined the effects of habitat on the predator functional response and foraging behavior of year-1 blue king crabs (predators) consuming year-0 conspecific crabs (prey). We hypothesized that (1) predator functional response, predation rates, and predator foraging behavior would vary with habitat; and (2) crypsis would increase with predator presence. Our results provide information that could help explain recruitment variability of some populations and help develop release strategies for stock enhancement efforts.

## Methods

### Ethics statement

Ethical approval for this research was not required by any federal, state, or international law because the animals used were invertebrates and therefore not covered. The transportation and field collection of the animals was authorized by the Alaska Department of Fish and Game (Fish Resource permit numbers CF-10-110, CF-11-012, CF-11-118, and CF-12-026).

### Experimental animals

Blue king crabs were cultured using established hatchery rearing techniques [41]. Ovigerous female blue king crabs were captured using baited commercial pots near Saint Matthew Island, Alaska, USA during 2010 and 2011. Larvae were cultured in cylindrical tanks until the first juvenile instar stages, at which point they were transferred to separate tanks with flow-through ambient seawater and held in populations. Bundles of gillnet were added to tanks to provide structure and minimize cannibalism [42]. Year-0 crabs were fed a combination of frozen *Artemia* (Brine Shrimp Direct, Ogden, Utah, USA), frozen bloodworms (Brine Shrimp Direct, Ogden, Utah, USA), frozen Cyclop-eeze (Argent Laboratories, Redmond, Washington, USA; Use of trade names does not imply endorsement by the National Marine Fisheries Service, NOAA), and a gel diet of “Gelly Belly” enhanced with Cyclop-eeze powder (Florida Aqua Farms, Inc., Dade City, Florida, USA) and walleye pollock (*Theragra chalcogramma*) bone powder (U. S. Department of Agriculture, Agricultural Research Service, Kodiak, Alaska, USA) twice per week. Year-1 crabs were held in individual enclosures to eliminate cannibalism [45] and were fed the same as above with the addition of frozen fish and squid. Food was provided to excess. Experiments were conducted in January and February 2013, approximately 19 months post-settlement for predator (year-1) crabs and approximately 7 months post-settlement for prey (year-0) crabs. Prey crabs were (average ± SE) 3.0±0.1 mm carapace width (CW) including spines, and predator crabs were 16.9±1.2 mm carapace length (CL) (range: 13.3 to 21.8 mm CL).

### Experimental apparatus and protocol

To determine the effect of habitat on the predator functional response, we performed predation trials in two different habitats at five prey densities. Predation trials were conducted in plastic containers 31×20×24 cm (L×W×H), held within a larger tank 170×90×30 cm (L×W×H) on a daily light cycle of approximately 10 h light and 14 h dark. Each container had flow through seawater (∼0.5 L min^−1^) maintained at 5.5°C, which is representative of nearshore waters around the Pribilof Islands [46]. Seawater entered the containers near the surface and exited through two holes (7.6 cm diameter) cut in opposite sides that were covered with 1 mm mesh. The bottom of each container was covered with 1 cm of sand collected from a local Kodiak Island beach and sieved through a 1 mm mesh screen. Some containers contained equal amounts of shell on top of the sand as substrate. Shells were a mix of clam valves, 65.1±2.6 mm (average ± SE) shell length (range: 42–102 mm, n = 30), collected from a local beach. We varied density at 2, 5, 10, 18, and 25 crabs container^−1^ and fully crossed prey density with habitat type (sand, shell). Replication (in parentheses) for each treatment was: 2 crabs container^−1^ (sand: 6, shell: 15), 5 crabs container^−1^ (sand: 5, shell: 8), 10 crabs conainer^−1^ (sand: 5, shell: 5), 18 crabs container^−1^ (sand: 5, shell: 5), and 25 crabs container^−1^ (sand: 6, shell: 5). We performed at least five replicates in all treatments and increased the sample size for treatments with high variance. Four control trials were performed at each treatment without predators.

Preliminary trials were run to ensure that predator crabs were motivated to forage on year-0 blue king crabs in the experimental system and to estimate the time required to achieve predation on multiple prey crabs. Hunger levels of predator crabs were standardized by depriving them of food for 24 h prior to trials [47]. On the morning (0730 h) of a predation trial, prey crabs were transferred to experimental containers and allowed to acclimate to new surroundings for 15 min so that crabs could locate preferred microhabitats. Predator crabs were then added and allowed to forage for 6 h. At the end of each trial, the number of prey crabs visible on the substrate was noted. Predator crabs were then removed and the substrate was thoroughly searched for prey crabs to determine survival rates.

A second set of trials was conducted to assess predator activity in sand and shell habitat. Each container (described above) was equipped with an overhead video camera monitored from an adjacent room to assure that the tanks were undisturbed during trials. Predator crabs were added to containers and allowed to acclimate for 24 h prior to trials. On the morning (0930 h) of a foraging trial, ten randomly selected prey crabs were placed in containers holding predators and video recording began. Predator crabs were allowed to forage for 120 min. There were five replicates of each habitat treatment. Video recordings of trials were subsampled for analysis. In each of twenty randomly selected minutes, predator crab behavior was classified as either 1) motionless (zero movement); 2) walking (moving laterally); or 3) foraging (consuming prey or repeated movements of chelae from the substrate to the mouth). The percentages of each activity were averaged to obtain one value of each behavioral parameter per predator crab.

### Analyses

We used maximum likelihood to fit the number eaten to:

Type I: 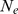  =  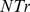


Type II: 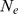  =  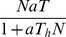


Type III: 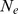  =  
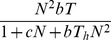


functional response models, where 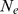 is the number of prey eaten, *N* is the number of prey available, *T* is the time available for foraging, *r* is the predation rate under a type I functional response, *a* is the instantaneous attack rate (or encounter rate) which describes how frequently a predator attacks (or encounters) a prey item, 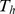 is the handling time which is the time it takes a predator to subdue and consume a prey item, and *b* and *c* are components of the instantaneous attack rate in a type III functional response [14], [15]. We assumed a binomial distribution of errors [48]. The data in sand and shell were fit separately and we calculated the Akaike's information criterion corrected for small sample size (AIC_c_) for each model and ranked them for each habitat type. Models with a ΔAIC_c_ of less than two were considered to explain the data equally well [49].

In trials with shell habitat, a crab crypsis index was calculated by dividing the number of prey crabs not visible at the end of the experiment (i.e., cryptic individuals) by the total number of live prey crabs. The assumption that crabs not visible were located within the shell material and were displaying a cryptic behavior was confirmed at the end of each trial. In sand habitat, the prey crabs did not bury themselves, were visible at all times, and were not cryptic. As such, we did not calculate a crab crypsis index in sand habitat. We used ANCOVA and regression analyses to determine differences in crypsis with and without predators with density as a covariate. To determine the effect of predator size on maximum predation rate, we analyzed the number eaten at the highest density (25 container^−1^) with a fully-crossed two-way ANCOVA with habitat as a factor and predator size (carapace length) as a covariate. We compared predator activity between sand and shell habitats for each behavior (motionless, walking, foraging) using t-tests. The assumption of homogeneity of variance was verified with a Levene's test and the assumption of normality with a Shapiro-Wilk's test. Statistics were done in SYSTAT 12.00.08 (Chicago, Illinois USA), R 2.9.2 (Vienna, Austria), and SigmaPlot 12.0 (San Jose, California).

## Results

### Predator functional response

**Figure 1:**
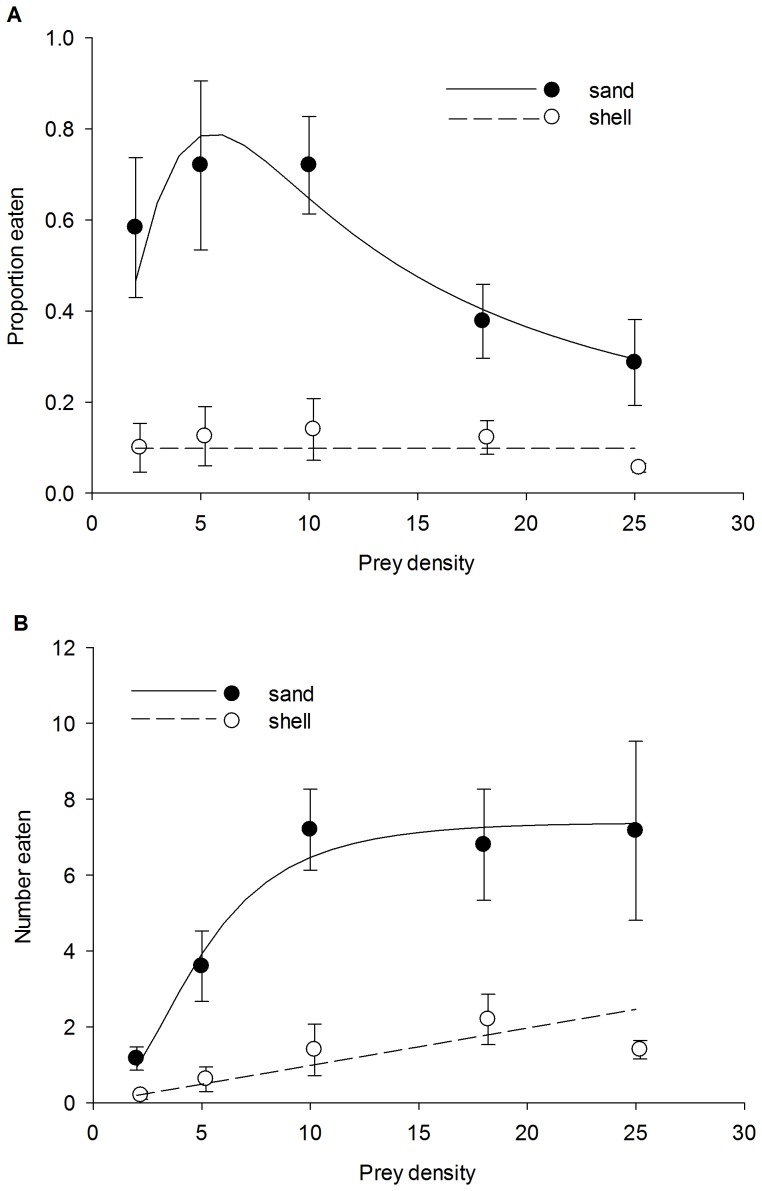
Functional response of year-1 blue king crabs (*P. platypus*) to year-0 blue king crabs density in sand (closed circles) and shell (open circles) habitat: (A) proportional predation and (B) number of prey crabs eaten. Points are the average (± SE) at each density and are offset slightly. Lines represent the best fit functional response model for each habitat. Parameter estimates (± SE) for sand are: *b* = 0.039 (0.023), *c* = −0.068 (0.19), *T_h_* = 0.84 (0.19), and for shell are: *r* = 0.016 (0.003).

In control trials, overall prey crab recovery was 100% indicating cannibalism within the year-0 cohort did not occur. In sand, the type III functional response model was best supported by our data (Fig. 1A, Table 1). Predator crabs appeared to reach satiation at approximately seven prey crabs (Fig. 1B). In shell, the data were unable to differentiate among the functional response models (Table 1), likely because of low predation rates at all prey densities (Fig. 1B). Because the type I functional response had the lowest AIC_c_ and is the most parsimonious model, we present and draw inferences from that model.

**Table 1:** Ranking of functional response models in sand and shell habitats using AIC_c_.

	Model	K	ΔAIC_c_	Likelihood	AIC_c_ Weights
Sand	Type I	1	33.38	0.00	0.00
	Type II	2	6.53	0.04	0.04
	Type III	3	0.00	1.00	0.96
Shell	Type I	1	0.00	1.00	0.43
	Type II	2	0.39	0.82	0.36
	Type III	3	1.41	0.49	0.21

### Prey crypsis, predator size, and predator behavior

**Figure 2:**
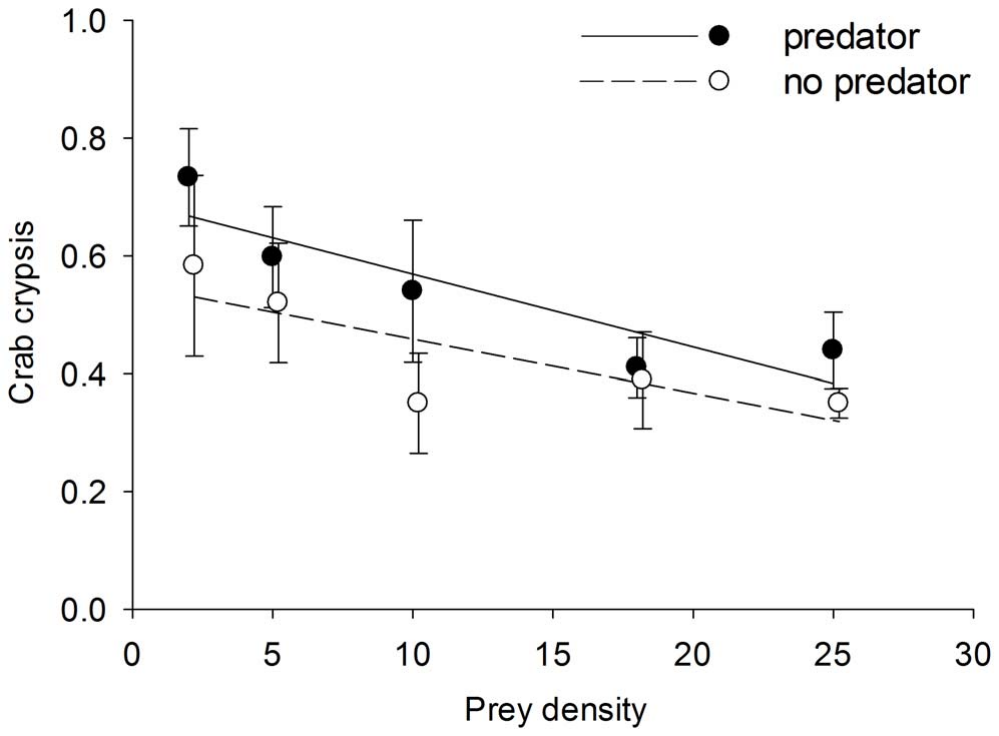
Average (± SE) prey crab crypsis index at five densities with (solid circles) and without (open circles) predator presence. The lines represent linear regressions for when predators were present (closed circles; crypsis = 0.732−(0.0134×density), R^2^ = 0.172) and absent (open circles; crypsis = 0.551−(0.00971×density), R^2^ = 0.106).

**Figure 3:**
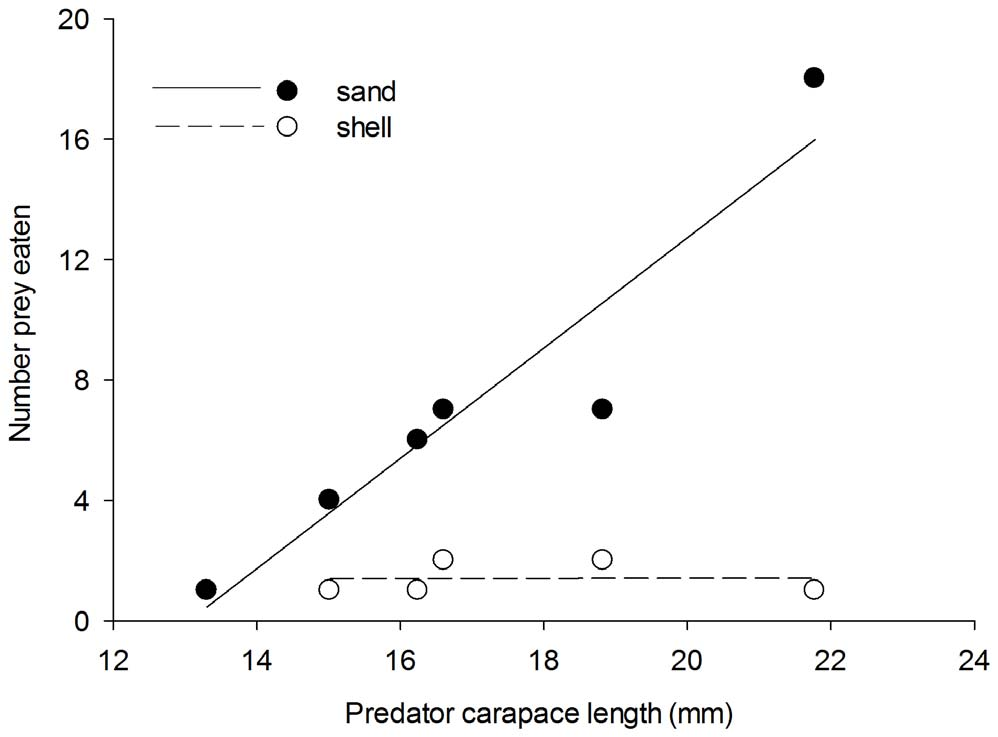
The number of prey crabs eaten as a function of predator size (carapace length) in sand (closed circles) and shell (open circles) habitat. Lines represent linear regression. Equations are: 1) sand: prey eaten = −23.926 + (1.883×carapace length), R^2^ = 0.895; and, 2) shell: prey eaten  = 1.373 + (0.00155×carapace length), R^2^ = 0.0000568.

**Figure 4:**
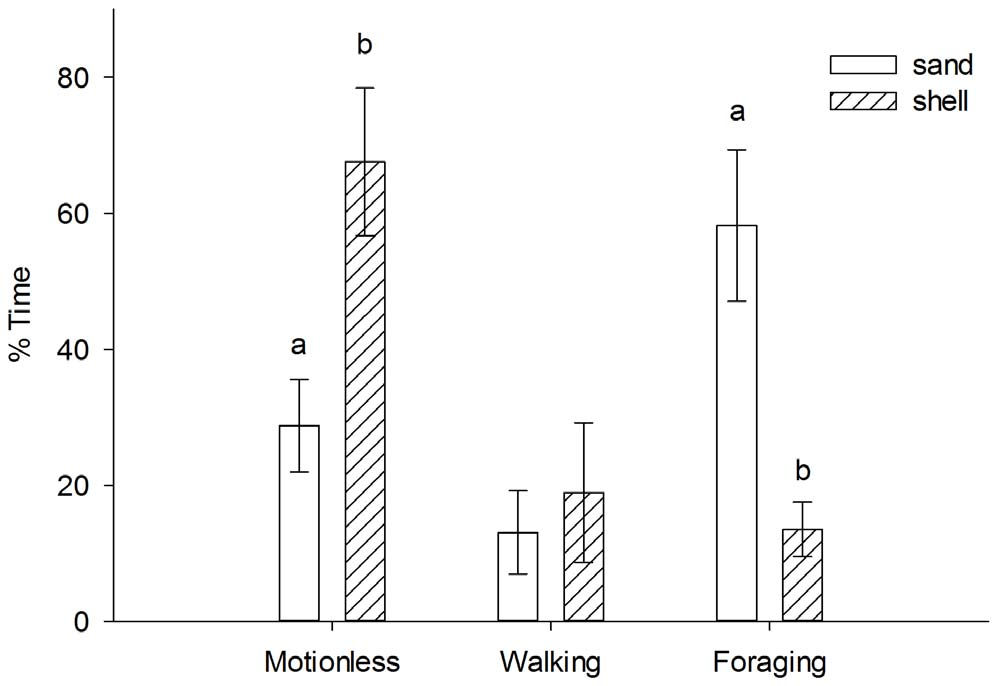
Average (± SE) predator crab activity in sand and shell habitat. We define “motionless” as crabs not moving, “walking” as crabs moving laterally, and “foraging” as crabs consuming prey or crabs with repeated movements of chelae from the substrate to the mouth. Different letters indicate statistical significance between habitats for each behavior (t test, p≤0.05).

Crab crypsis in shell was generally high, decreased with prey density, and was marginally affected by predator presence (Fig. 2, Table 2). The prey density × predator presence interaction was not significant indicating homogeneity of regression slopes (Table 2). Because there was no interaction effect and the main effect of predator presence was marginally non-significant, we reran the ANCOVA without the interaction term. Main effects were then significant for density (*df* = 1, F = 9.823, *p* = 0.003) and predator presence (*df* = 1, F = 5.071, *p* = 0.028; Table 2). Overall, crab crypsis was 34% higher in the presence of predators compared to without predators. The number of prey eaten at the highest density (25 crabs container^−1^) showed a significant predator size × habitat interaction, indicating slopes were unequal (ANCOVA, Table 2). As such, sand and shell habitats were analyzed separately with linear regression. The number of prey crab eaten increased with predator size in sand (p = 0.004), but not in shell (p = 0.990) (Fig. 3). Behavior of predator crabs varied between habitats. Predator crabs spent significantly more time motionless (t-test, t = −3.031, df = 8, p = 0.016) and less time foraging (t-test, t = 3.778, df = 8, p = 0.005) in shell compared to sand (Fig. 4).

**Table 2:** ANCOVAs for crypsis and the number of prey eaten.

	Source of variation	SS	*df*	MS	*F*	*p*
Crypsis	Density	0.529	1	0.529	8.752	**0.004** a
	Predator presence	0.195	1	0.195	3.235	0.077^b^
	Density × predator presence	0.014	1	0.014	0.224	0.638
	Error	3.443	57	0.060		
Prey eaten	Predator size	58.285	1	58.29	21.72	**0.002**
	Habitat	35.807	1	35.81	13.35	**0.008**
	Predator size × habitat	58.088	1	58.09	21.65	**0.002**
	Error	18.781	7	2.68		

Bold indicates statistical significance (α≤0.05).

a *p* = 0.003 without interaction term included in the model.

b *p* = 0.028 without interaction term included in the model.

## Discussion

Our results demonstrate that early benthic phase blue king crabs are cannibalistic between cohorts and that habitat mediates the functional response of year-1 individuals. Predation rates are generally limited by handling time (i.e., the time required to pursue, subdue, and consume prey) in a type II functional response, and limited by encounter rate at low prey densities in a type III functional response [16]. Because prey crabs were not cryptic in sand, the type III functional response is surprising. Typically, cryptic species yield a type II functional response in simple habitats, which sometimes changes to a type III in complex habitat [13], [22], [50], but can remain a type II [17], [20]. Two possible mechanisms may have led to a type III functional response in our experiment. Either prey crabs were more effective at avoiding predators at low densities, or predators reduced the time spent foraging at low prey densities.

The physical architecture of stacked shell debris is an efficient refuge habitat for early benthic phase blue king crabs. A type I functional response is common in “sit-and-wait” predators, where probability of prey encounter increases linearly with prey density [51], [52]. Yet, even at the highest density, prey consumption was low, likely because shell structure decreased predator-prey encounter rates. The relatively flat crevice matrices allow for more interstitial spaces per volume compared to other structurally complex habitats, such as cobble [33]. Additionally, the blue king crab light, mottled coloration is an extremely effective visual camouflage, while the relatively smooth, flat carapace is especially suited for physical crypsis in shell [33]. As such, even a low volume of shell debris provides adequate crevice space for multiple individuals.

In many species, predator avoidance behavior depresses food intake, which can reduce growth and overall fitness causing a trade-off between foraging efficiency and predator avoidance, as both are seldom maximized simultaneously [53]–[57]. Unless a refuge habitat provides adequate food resources, individuals must make a behavioral decision to improve this trade-off. Early benthic phase red king crabs seek habitats that provide both structural complexity and foraging opportunities in order to optimize growth and survival [58]. Because prey density was relatively high in our experimental mesocosms compared to natural systems [29], the reduced foraging activity by year-1 predator crabs in shell may be an attempt to optimize predator avoidance while food resources are abundant, but foraging activity would likely increase with time as hunger levels rise. Newly emerged crab spiderlings (*Misumena vata*) display similar patterns of differential foraging strategies, where initial search time is reduced in structurally complex substrate but increases over time [53]. Alternatively, shell may provide such an effective refuge for year-0 crabs that increased foraging by year-1 crabs does not optimize the trade-off between foraging and predator avoidance.

The behavioral response of year-0 prey crabs to the presence of predators was not surprising. Early benthic phase red king crabs respond to conspecific and fish predators in experimental tanks by increasing affinity for or moving to complex structures [36], [47], [59] and individuals with prior predator experience display improved crypsis [60]. Yet, early benthic phase blue king crabs display high levels of crypsis regardless of the presence of red king crab predators in similar laboratory studies [33]. In combination with their modest spination, the generally high levels of crypsis may reflect a strong reliance on spatial separation from predators, rather than pronounced spines or aggressive displays as a predator defense.

Our results have direct implications for blue king crab stock enhancement. Predation by a variety of species will likely be the first challenge hatchery-cultured individuals face once released in the wild, thus selecting release habitats with adequate refugia is needed to optimize survival. Specifically, shell material will likely be the most effective habitat for blue king crab releases because of its ample crevice spaces. These results suggest that moderate to high stocking densities may allow for good survival from conspecific predators, but perhaps not other predator species. For example, localized dense blue king crab populations following a release may attract demersal fish predation. As such, predator suites must be considered when evaluating potential release sites. For blue king crab hatchery culture, optimal size for release is unknown and may require long-term hatchery rearing. Different cohorts should be reared separately with complex structures to reduce cannibalism and maximize hatchery production.

We demonstrate that blue king crabs display inter-cohort cannibalism and that shell changes predator functional response by reducing foraging behavior. Although associations of early benthic phase blue king crabs with shell material is somewhat well documented in the field [29]-[31] and laboratory [28], our study illustrates its importance for reducing vulnerability to predators [32], [33]. Given the importance of structural complexity, the extent and distribution of shell material may influence recruitment success of some populations. Field surveys indicate a broad distribution of shell habitat around the Pribilof Islands [29], thus habitat availability alone cannot explain the extremely low abundance of the Pribilof stock and the brief recovery of the Saint Matthew population. Blue king crab population abundances around the Pribilof and Saint Matthew Islands are probably influenced by a combination of ecosystem-level processes such as Allee effects, differential predation, and larval advection, among others. Future studies should compare benthic species assemblages around the Pribilof Islands and Saint Matthew Island to identify possible population bottlenecks. Further, examining spatial connectivity between ovigerous females and optimal settling habitat in both locations would help link climatic conditions and female distribution to recruitment success.
